# Supplementary material for: Automating parameter selection to avoid implausible biological pathway models
Source: NPJ Syst Biol Appl. 2021 Feb 23;7:12. doi: 10.1038/s41540-020-00167-1 (PMC7902638; doi:10.1038/s41540-020-00167-1)
Supplement: Supplementary file 1 — Supplementary Information [file 41540_2020_167_MOESM1_ESM.pdf]

Supplementary Information: Automating parameter selection to avoid implausible biological pathway models

Chris S. Magnano and Anthony Gitter

Supplementary Figures

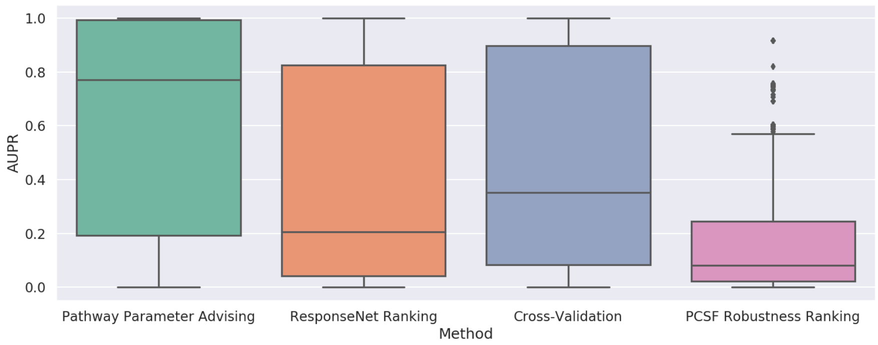

Supplementary Figure 1. Performance of parameter selection methods on avoiding implausible networks aggregated for all considered 10,000 plausibility criteria. Boxplots are filled in from the first to third quartiles with a line at the median, and whiskers representing 1.5 times the interquartile range. AUPR is shown for all 12 NetPath test pathways and 4 pathway reconstruction methods.

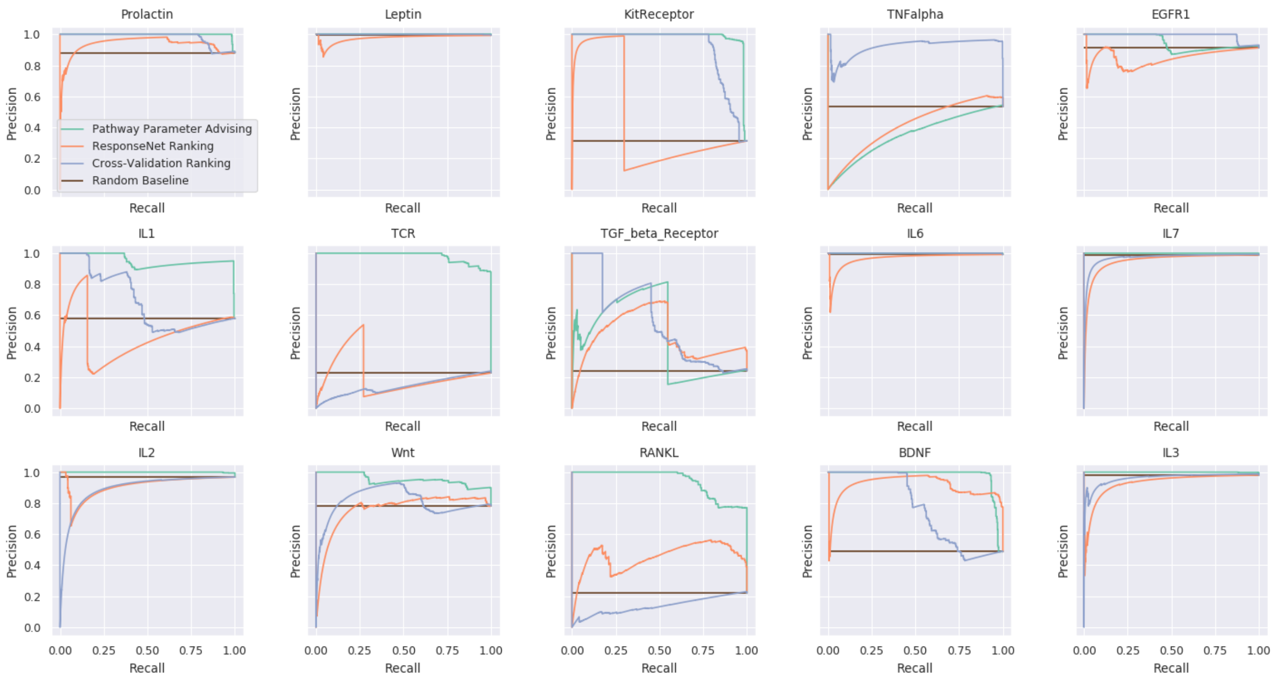

Supplementary Figure 2. All PR curves for implausible pathway detection using different parameter ranking schemes for pathways created using PathLinker.

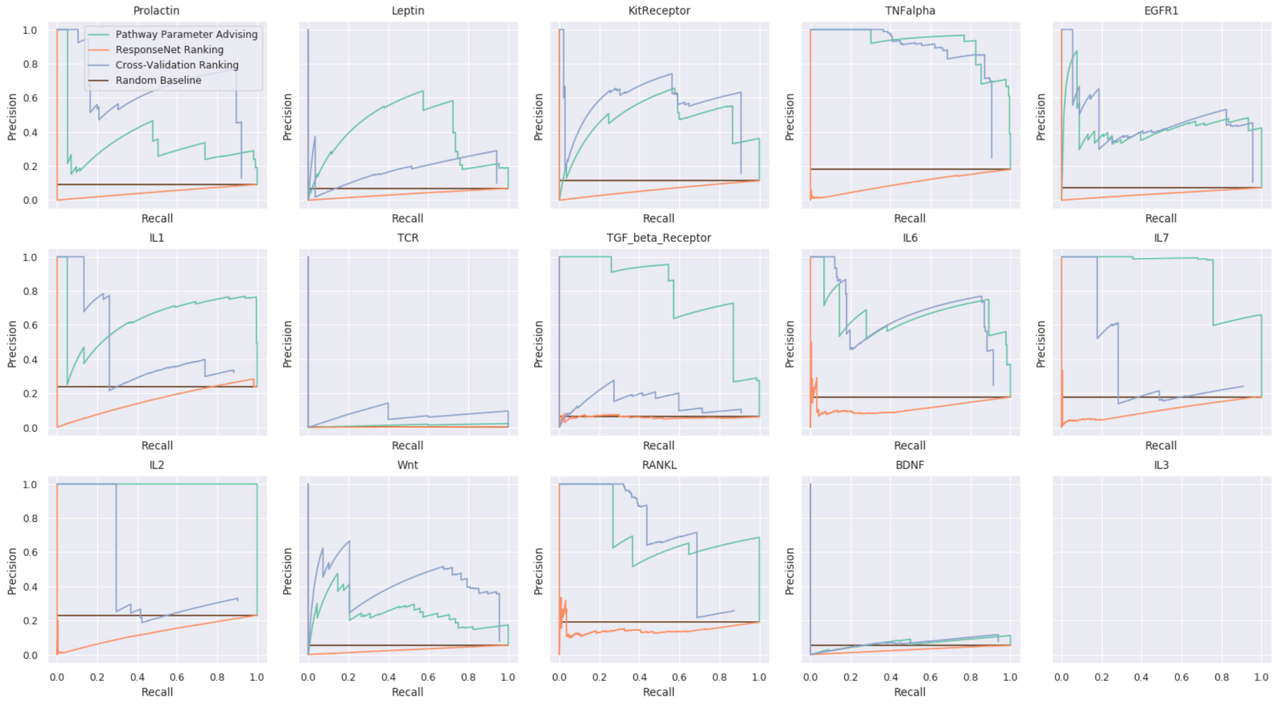

Supplementary Figure 3. All PR curves for implausible pathway detection using different parameter ranking schemes for pathways created using minimum-cost flow. The blank panel for IL3 indicates that no reconstructed pathways were plausible.

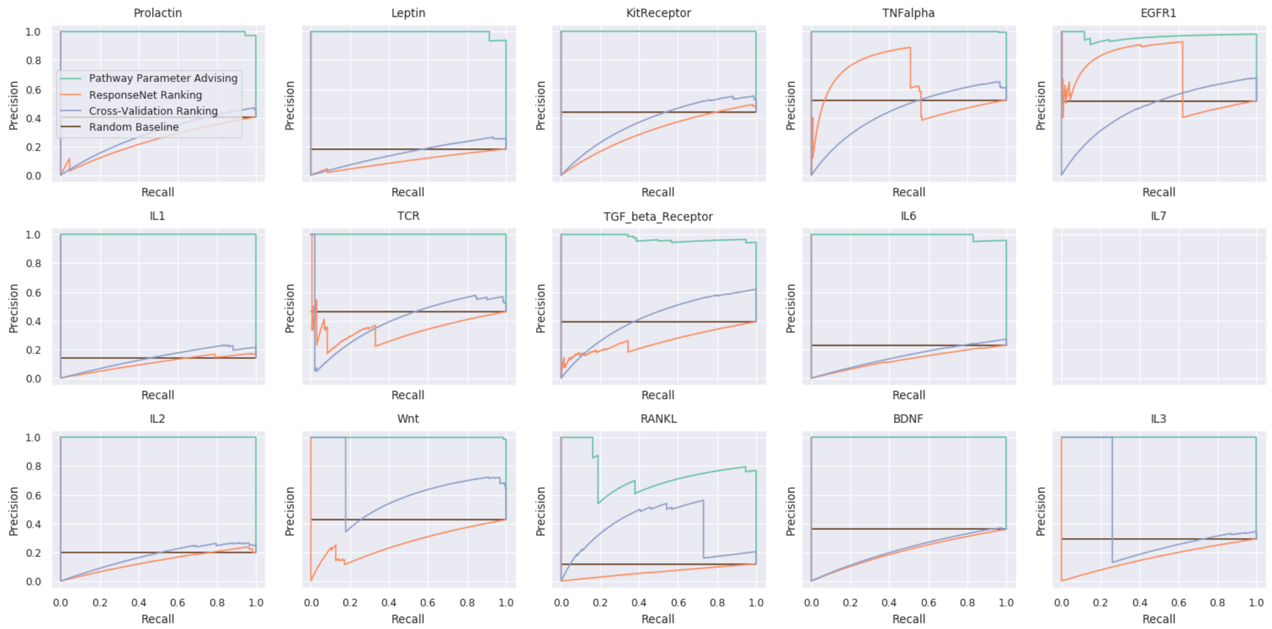

Supplementary Figure 4. All PR curves for implausible pathway detection using different parameter ranking schemes for pathways created using PCSF. The blank panel for IL7 indicates that no reconstructed pathways were plausible.

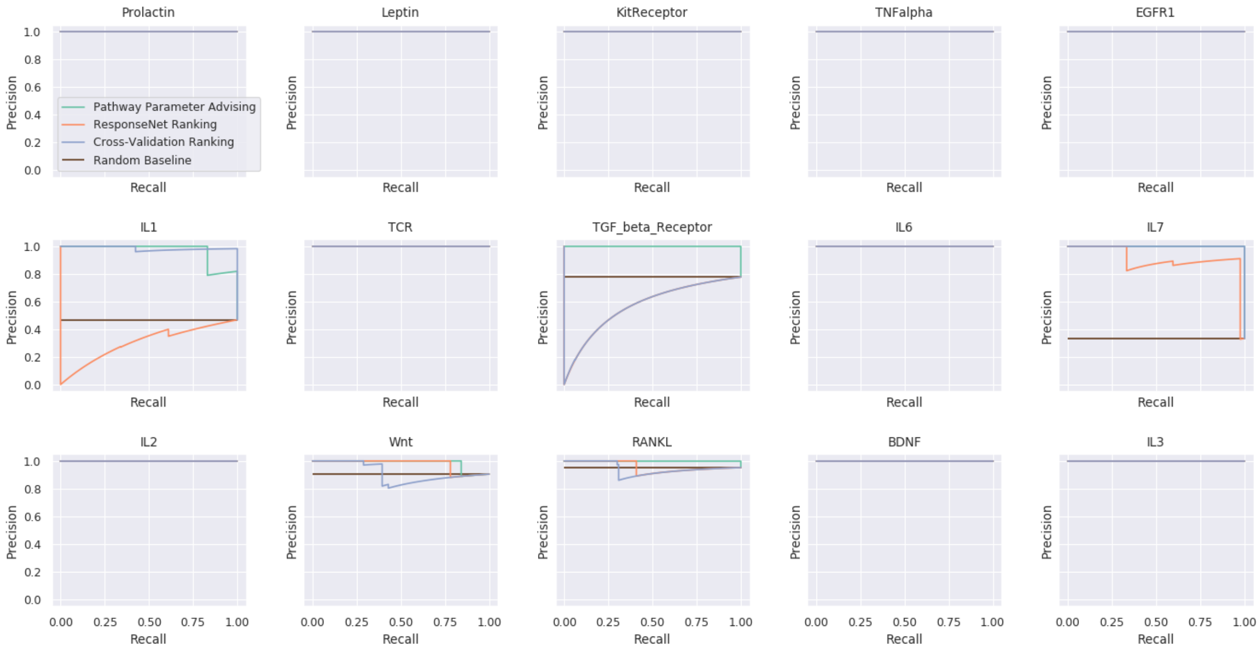

Supplementary Figure 5. All PR curves for implausible pathway detection using different parameter ranking schemes for pathways created using NetBox. In 10 cases every reconstructed pathway met the plausible pathway criteria.

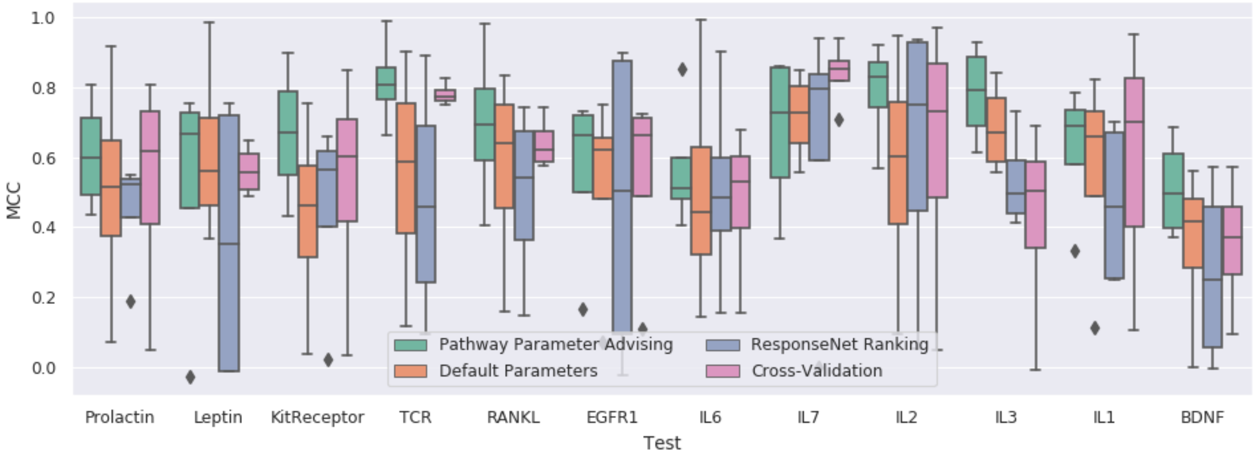

Supplementary Figure 6. Adjusted MCC values for different parameter selection methods in the NetPath pathway reconstruction. Boxplots are filled in from the first to third quartiles with a line at the median, and whiskers representing 1.5 times the interquartile range.

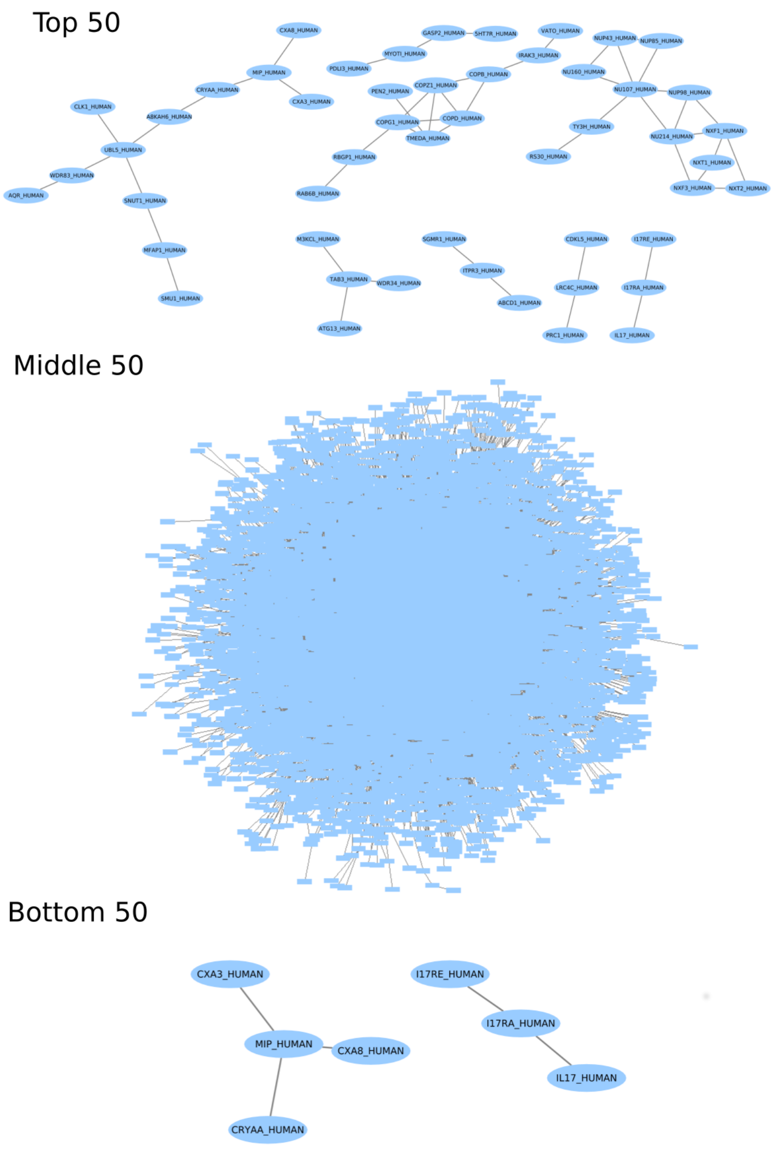

Supplementary Figure 7. Influenza host factor pathways created by ensembling PCSF runs. The resulting pathways from the top 50, middle 50, and bottom 50 parameter settings as ranked by pathway parameter advising. All connected components over 3 nodes are shown.

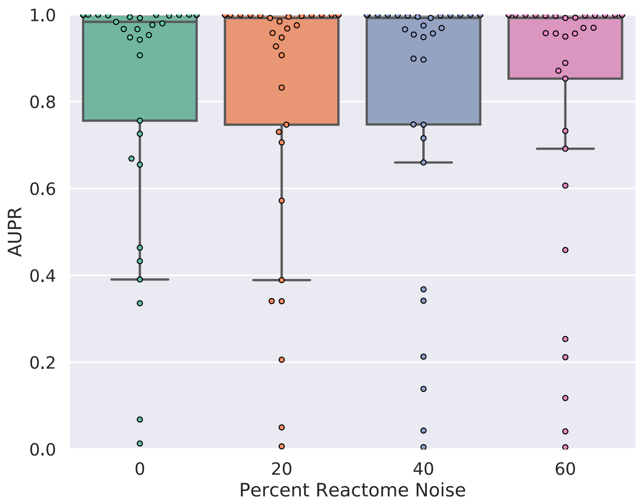

Supplementary Figure 8. Performance of pathway parameter advising when noise is added to reference pathways. Noise was added to all Reactome pathways as described in Section "Evaluating the Ranking Metric", and AUPR was calculated for implausible pathway detection as described in Section "Implausible Pathway Detection" for 12 NetPath test pathways. Adding noise to the set of reference pathways resulted in almost no change to the distribution of AUPR scores over all tests. Boxplots are filled in from the first to third quartiles with a line at the median, and whiskers representing 1.5 times the interquartile range.

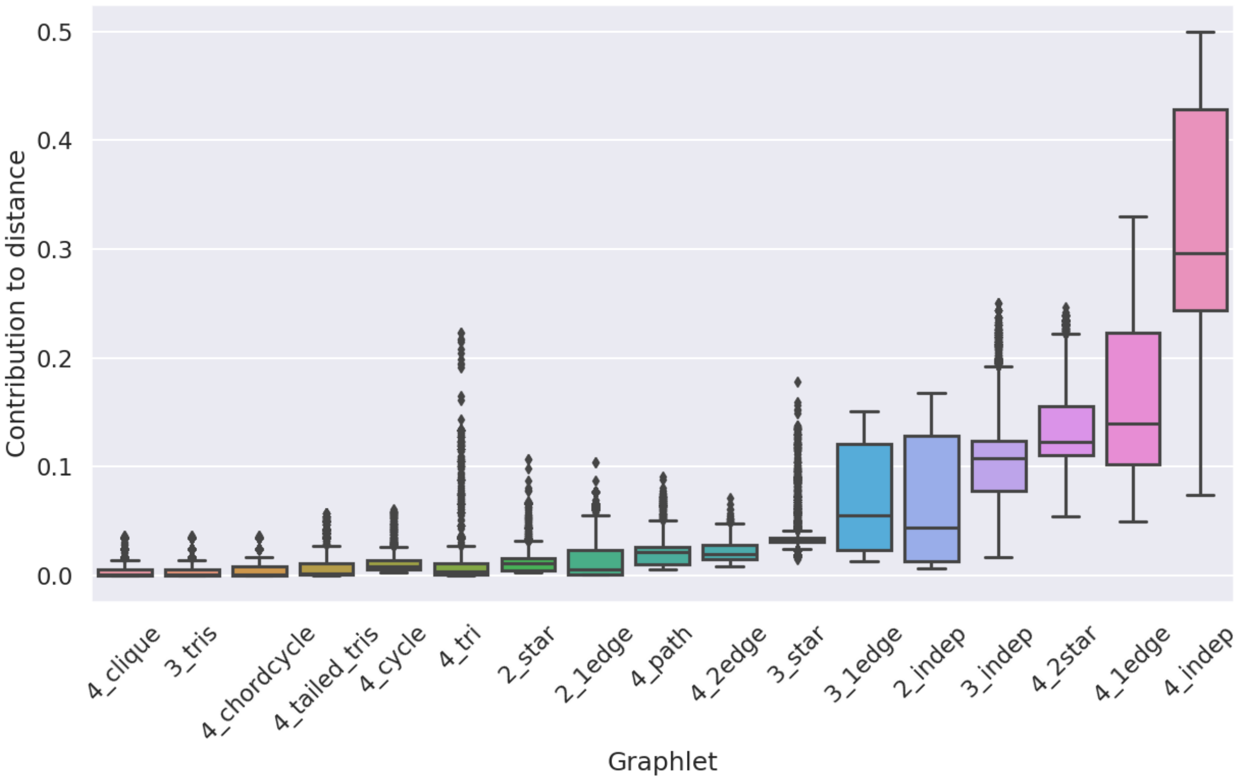

Supplementary Figure 9. Contribution of each of the 17 graphlets to graphlet distance across the NetPath validation pathways Wnt, TNF alpha, and TGF beta and 4 pathway reconstruction algorithms. Graphlets are labeled as according to [40]. The 4 disconnected nodes graphlet, 4_indep, has a median contribution of about 30% of the GFD. Boxplots are filled in from the first to third quartiles with a line at the median, and whiskers representing 1.5 times the interquartile range.

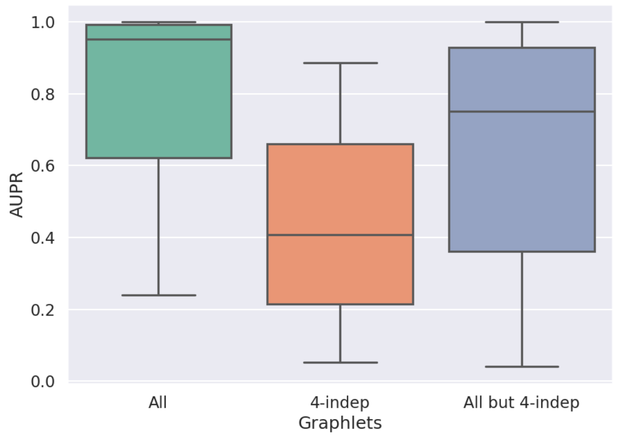

Supplementary Figure 10. Performance of parameter selection methods on avoiding implausible networks using different sets of graphlets: all graphlets (All), only the 4 disconnected node graphlet (4-indep), and all graphlets except the 4 independent node graphlet (All but 4-indep). Boxplots represent the distributions of the AUPRs aggregated for 4 pathway reconstruction methods and the NetPath validation pathways Wnt, TNF alpha, and TGF beta. Using all graphlets yields the best performance. Removing the 4 disconnected node graphlet slightly lowers the AUPR, and only using the 4 disconnected node graphlet results in a large performance decrease. This suggests that our ranking method is highly dependent on graphlets other than the 4 disconnected node graphlet. Boxplots are filled in from the first to third quartiles with a line at the median, and whiskers representing 1.5 times the interquartile range.

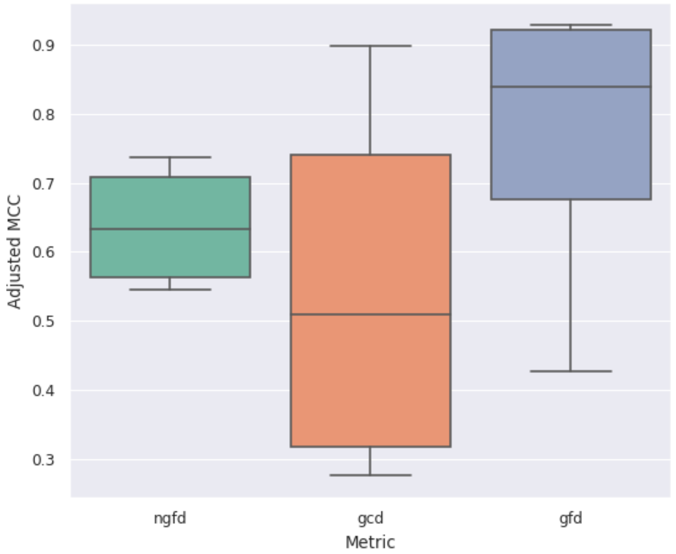

Supplementary Figure 11. Examining the effect of different graphlet-based distance metrics on the adjusted MCC of pathway reconstruction. Reconstructions were performed on the NetPath validation pathways Wnt, TNF alpha, and TGF beta across the 4 pathway reconstruction algorithms. We examined 3 graphlet-based metrics for pathway parameter advising: normalized graphlet frequency distance (NGFD), graphlet correlation distance (GCD), and graphlet frequency distance (GFD). For NGFD, we wanted to explore a metric that takes advantage of all reconstructed pathways being sub-networks of the same interactome. Thus, we normalized all graphlet frequencies by the corresponding graphlet's frequency in the interactome. We also explored GCD, which measures the correlation between connected graphlets in a pathway [41]. This creates a metric that is solely focused on local topology and has minimal information about pathway size or other global topological properties. Adjusted MCC was calculated the same way as in Section "Quality of NetPath Pathway Reconstruction". Boxplots are filled in from the first to third quartiles with a line at the median, and whiskers representing 1.5 times the interquartile range.

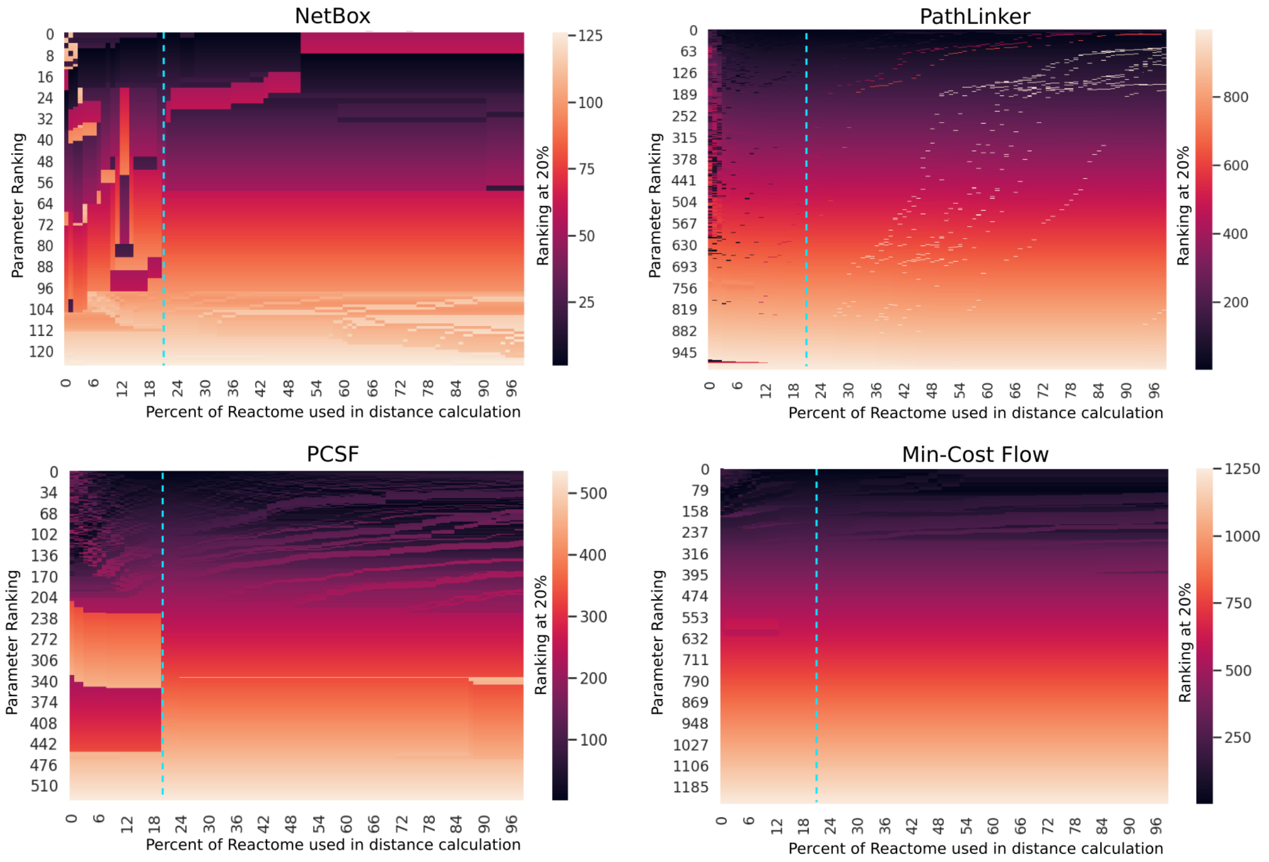

Supplementary Figure 12. Change in ranking order over different percent thresholds for calculating score in pathway parameter advising on the TGF beta receptor NetPath pathway. Heatmaps are colored based on the ranking at the chosen threshold, 20% (marked by a blue dashed line). While the parameter ranking is unstable for very small values, by 20% the ranking remains generally unchanged as the threshold continues to increase, as can be seen in the color gradient consistency in the right halves of the figures.

Supplementary Tables

Supplementary Table 1. All GO terms returned from GO-term enrichment with adjusted p-value < 0.05 using DAVID on all nodes in the influenza host factor pathway. The influenza host factor pathway was created using the top 50 pathways ranked by pathway parameter advising constructed using PCSF. The column Benjamini shows p-values corrected using the Benjamini-Hochberg procedure.

Term	Term Name	Count	Benjamini
GO:0006406	mRNA export from nucleus	9	9.07E-06
GO:0006409	tRNA export from nucleus	6	7.65E-05
GO:0010827	regulation of glucose transport	6	5.98E-05
GO:0019083	viral transcription	8	9.81E-05
GO:0007077	mitotic nuclear envelope disassembly	6	0.000157
GO:0075733	intracellular transport of virus	6	0.000277
GO:1900034	regulation of cellular response to heat	6	0.00159
GO:0006890	retrograde vesicle-mediated transport, Golgi to ER	6	0.00215
GO:0031047	gene silencing by RNA	6	0.00806
GO:0016925	protein sumoylation	6	0.00928
GO:0007601	visual perception	7	0.0137
GO:0016032	viral process	8	0.0175
GO:0006886	intracellular protein transport	7	0.0268
GO:0007062	sister chromatid cohesion	5	0.0408

Supplementary Table 2. The 9 KEGG pathways returned from enrichment using DAVID of all nodes in the influenza host factor pathway. The influenza host factor pathway was created using the top 50 pathways ranked by pathway parameter advising constructed using PCSF. The column Benjamini shows p-values corrected using the Benjamini-Hochberg procedure.

Term	Term Name	Count	Benjamini
hsa03013	RNA transport	10	4.00E-05
hsa05164	Influenza A	6	0.134
hsa03008	Ribosome biogenesis in eukaryotes	4	0.346
hsa05323	Rheumatoid arthritis	4	0.280
hsa03015	mRNA surveillance pathway	4	0.250
hsa05110	Vibrio cholerae infection	3	0.433
hsa00190	Oxidative phosphorylation	4	0.427
hsa04721	Synaptic vesicle cycle	3	0.455
hsa05120	Epithelial cell signaling in Helicobacter pylori infection	3	0.454
